# Daily high doses of atorvastatin alter neuronal morphology in a juvenile songbird model

**DOI:** 10.1371/journal.pone.0314690

**Published:** 2025-04-28

**Authors:** Shuk C. Tsoi, Alicia C. Barrientos, David S. Vicario, Mimi L. Phan, Carolyn L. Pytte

**Affiliations:** 1 CUNY Neuroscience Collaborative, Psychology and Biology Departments, The Graduate Center, City University of New York, New York, New York, United States of America; 2 Department of Psychology, Rutgers, The State University of New Jersey, Piscataway, New Jersey, United States of America; 3 Psychology Department, Queens College, City University of New York, Flushing, New York, United States of America; Pennsylvania State University, UNITED STATES OF AMERICA

## Abstract

Statins are highly effective and widely prescribed cholesterol lowering drugs. However, statins cross the blood-brain barrier and decrease neural cholesterol in animal models, raising concern that long-term statin use may impact cholesterol-dependent structures and functions in the brain. Cholesterol is a fundamental component of cell membranes and experimentally decreasing membrane cholesterol has been shown to alter cell morphology *in vitro.* In addition, brain regions that undergo adult neurogenesis rely on local brain cholesterol for the manufacture of new neuronal membranes. Thus neurogenesis may be particularly vulnerable to long-term statin use. Here we asked whether oral statin treatment impacts neurogenesis in juveniles, either by decreasing numbers of new cells formed or altering the structure of new neurons. The use of statins in children and adolescents has received less attention than in older adults, with few studies on potential unintended effects in young brains. We examined neurons in the juvenile zebra finch songbird in telencephalic regions that function in song perception and memory (caudomedial nidopallium, NCM) and song production (HVC). Birds received either 40 mg/kg of atorvastatin in water or water vehicle once daily for 2–3 months until they reached adulthood. We labeled newborn cells using systemic injections of bromodeoxyuridine (BrdU) and quantified cells double-labeled with antibodies for BrdU and the neuron-specific protein Hu 30–32 days post mitosis. We also quantified a younger cohort of new neurons in the same birds using antibody to the neuronal protein doublecortin (DCX). We then compared numbers of new neurons and soma morphology of BrdU + /Hu+ neurons between statin-treated and control birds. We did not find an effect of statins on the density of newly formed neurons in either brain region, suggesting that statin treatment did not impact neurogenesis or young neuron survival in our paradigm. However, we found that neuronal soma morphology differed significantly between statin-treated and control birds. Somata of BrdU + /Hu+ (30–32 day old) neurons were flatter and had more furrowed contours in statin-treated birds relative to controls. In a larger, heterogeneous cohort of non-birthdated BrdU-/Hu+ neurons, largely born prior to statin treatment, somata were smaller in statin-treated birds than in controls. Our findings indicate that atorvastatin may affect neural cytoarchitecture in both newly formed and mature neurons, perhaps as a consequence of decreased cholesterol availability in the brain.

## Introduction

Statins are potent inhibitors of cholesterol synthesis that effectively reduce plasma cholesterol synthesis by the liver, thereby reducing cardiovascular diseases and other health risks of high cholesterol [1,2]. In addition to peripheral synthesis, cholesterol is produced independently in the central nervous system, mainly by astrocytes and oligodendrocytes. Orally administered statins have been shown to pass into the brain and decrease brain cholesterol, both through passive transport of the more lipophilic statins as well as active transport of hydrophilic forms [cf. 3,4–9], cf. [10]. Brain cholesterol is regulated centrally, without feedback or supplementation from serum cholesterol [7,11–13], thereby raising questions about the impact of oral statins on cholesterol-dependent neural tissue and function [3,14–17].

Effects of statins on neural substrates are most notably recognized as neuroprotective, likely due in part to their strong anti-inflammatory properties independent of effects on cholesterol synthesis. Thus statins are broadly touted as an intervention for neurodegeneration in disease, traumatic brain injury, hemorrhage, epilepsy, and stroke [18–20]; but note cautionary review [21]. In reports of children with neural damage, statins may likewise be neuroprotective by suppressing inflammatory processes [22–24]. However, cognitive complaints by some statin users resulted a 2012 Food and Drug Administration warning that statins may induce cognitive dysfunction, including impaired memory and confusion [25,26, cf. 27]. Subsequent metareviews, based primarily on studies with subjects older than 60 years of age, report divergent results: summarizing conflicting findings [28], concluding that statin use does not increase cognitive decline in older adults [29,30], alternatively concluding that memory loss is a frequent side effect of atorvastatin [31] and finding that the association between neurocognitive disorders and statins (atorvastatin, simvastatin, and pravastatin) increases with age [32]. In sum, there is still no consensus.

It is clear, however, that in contrast to many studies of effects of statins in older populations and injured or diseased brains, very little research has investigated potential effects of statins in young and healthy brains–either through direct effects of statins on neural tissue or indirectly via a reduction in brain cholesterol or any number of intermediary products of the cholesterol synthesis pathway. The relevance of this question stems from FDA approval for seven statins (to date) for use in children as young as 8–10 years old who have a genetic risk for developing the autosomal dominant disorder familial hypercholesterolemia. Statins have also been recommended by the American Academy of Pediatrics to treat children without a genetic risk factor, but who nonetheless have high levels of cholesterol not responsive to diet or exercise. However, the supporting studies on efficacy and safety have not evaluated measures of brain health, instead focusing on toxicity, plasma hormone levels, metrics of sexual development, pharmacokinetics, and school performance [33–41].

It is estimated that almost a quarter of the body’s total cholesterol content is in the brain, making it the most cholesterol-rich organ in the body [11,42]. The majority of the brain’s cholesterol content (~70%) is found in myelin with the remainder occurring in cell membranes [43–45]. The plasma membrane incorporates cholesterol into specialized subdomains called lipid rafts [46], which play a critical role not only in membrane structure and organization, but also membrane dynamics and interactions with transmembrane proteins [47–49]. Cholesterol also plays a role in the transport of proteins and paracrine signaling [11,12,42,45]. *In vitro* studies have demonstrated extensive effects of decreased cholesterol availability on neuronal structure including impaired receptors, damaged organelles, compacted nuclei with condensed chromatin, a decrease in neurite density, fragmented neurites, and spine shrinkage [50–54]. Exposure to statins *in vitro* has also been shown to alter neuronal function, resulting in decreased synaptic vesicle release and reduced synapse densities [55] and to induce cell death of both cortical neurons [51,52] and cerebellar neurons [56,57]. In addition to effects on neurons, statin exposure has been shown to alter cellular morphology and decrease survival of astrocytes [50], microglia [56], and oligodendrocytes [57] *in vitro.* Interestingly, no work has yet determined whether systemic administration of statins affects neural cell structures *in vivo* in the healthy brain.

Regions of the brain that undergo life-long neurogenesis, with a continual demand for new membrane production, may be particularly sensitive to alterations in brain cholesterol. Notably, there is some direct evidence that the level of available cholesterol impacts the rate of new neuron production: Neural stem and progenitor cells are dependent on endogenous cholesterol synthesis for survival [58] and cholesterol derivatives (oxysterols) have been shown to function as ligands, directly contributing to neurogenesis and neuronal survival during embryonic brain development [59]. In addition, genetically reducing the activity of a cholesterol synthesis enzyme caused abnormal neural progenitor fate determination, among other alterations of embryonic neurogenesis in utero [60]. Thus, dynamics of post-embryonic neurogenesis and neuron formation throughout life, and cognitive functions that rely on new neurons, may be distinctly vulnerable to statin exposure. In addition, mature neurons continue to cycle plasma membrane cholesterol, requiring local cholesterol availability.

Therefore, as a first step toward understanding the effects of statin use on healthy neural tissue *in vivo*, we used a juvenile zebra finch model to determine whether long-term oral atorvastatin, a moderately lipophilic statin, alters the density and soma cytoarchitecture of neurons born during statin exposure. We also sought to determine whether statins may have an effect on soma structure of neurons already established at the time of statin exposure.

## Materials and methods

### Animals

All work was approved by the CUNY Queens College and Rutgers University Institutional Animal Care and Use Committees. We restricted our study to males because we wanted to decrease inherent variability in our morphological measurements by limiting the sample to known cell types, which have been established in the song system nucleus HVC in males [61,62]. HVC receives new neurons throughout adulthood and is necessary for song learning and production. It is thus a region that may be sensitive to, or reflect, potential behavioral effects of statins, consistent with our goal of establishing a juvenile model system. Female HVC is substantially smaller than in males, and more importantly for our purposes, neuronal cell types in female HVC have not yet been characterized and HVC is not used for song production [63–65]. Opportunistically, we also used the higher-order auditory region of the caudomedial nidopallium (NCM) in the same male birds to quantify new neurons in both NCM and HVC to determine whether statins alter new neuron production or recruitment to these regions.

Zebra finches were bred and housed in the Rutgers University vivarium. The birds were kept on a 12 hr light:12 hr dark cycle with *ad libitum* access to food and water at a room temperature of 21–28°C. Birds were bred in single-family cages or large double-family cages in a large aviary room, in auditory and visual contact with other birds. Zebra finches fledge at approximately 14 days of age, feed independently at approximately 30 days, and are considered adults at about day 90. Birds were moved from family cages to individual housing in sound-attenuated chambers at 35–40 days of age. Individuals were age-matched and sibling-matched across treatment groups such that pairs of males in each clutch were assigned to the statin-treated or control group with an additional sibling male added to the control group. Birds acclimated to isolate housing in sound attenuated chambers for 38–40 days prior to the first 5-bromo-2’-deoxyuridine (BrdU) injection. Because isolate housing without a song model in birds of this age impacts neurogenesis [66], we standardized the song learning environment with a widely used operant/housing protocol. In this, both control and statin-treated birds were exposed to the model song termed “Samba” in response to key pecks (following [67]).

### Timeline

We used a wide age range for the start of the statin/vehicle treatment in order to test whether the bird’s age at the start of statin treatment or the duration of exposure impacts densities of new neurons in HVC or NCM, or morphological measurements of neurons in HVC. At the start of the experiment, juvenile zebra finches were 18–49 days old (mean = 36 days, SEM = 2.67) and were given atorvastatin or water vehicle. Treatments continued for 62–92 days (mean = 74 days of treatment, SEM = 2.645) until perfusion at ages 107–111 days old.

In contrast, we sought to limit individual variation in neurogenesis due to factors not related to statin treatment. Because new neurons added to HVC decline with age, we used a narrow rage of bird ages at the time of cell birthdating with BrdU. Birds were 75–79 days old at the first BrdU injection and all birds were perfused 30 days after the last injection to compare densities of same-aged BrdU+ labeled neurons (30–32 days after mitosis). Birds were 107–111 days old at the time of perfusion. Thus, all BrdU+ labeled cells were born while the birds were exposed to statins or vehicle. At the time of BrdU injections, birds had received daily doses of statin or vehicle for 30–60 days.

### Atorvastatin treatment

Experimental birds (n = 6) were given daily doses of 40 mg/kg of atorvastatin (Lipitor®, Pfizer) dissolved in 50 μl of water, administered by pipette to the side of the beak. Controls (n = 7) were given the same daily volume of water vehicle in the same manner to control for the stress of handling.

Forty mg/kg of atorvastatin is a higher dose than used clinically, yet lower than the commonly used dose of 50 mg/kg in studies with rodent models, e.g., [8]. The Federal Drug Administration (FDA)-approved atorvastatin dose is 10–80 mg/day for adults and 10–20 mg/day for children, based on a standard child weight of 20 kg (44 lb), i.e., a child dose range of = 0.5 mg/kg -1.0 mg/kg. FDA animal model pharmaceutical conversion rates are calculated by animal size (i.e., body surface area to volume ratios) across animal models. Given that zebra finches are not listed in FDA equivalence charts, we used the mouse conversion factor as an approximately equivalent body size, which requires multiplying the animal dose by 0.08 as an approximation of the human equivalence dose. Thus our dose of 40 mg/kg, multiplied by 0.08, is equivalent to a human dose of 3.2 mg/kg. Our dose is therefore (3.2 divided by 0.5) 6.4 times the lower end and (3.2 divided by 1.0) 3.2 times the higher end of the child equivalent dose (Food and Drug Administration, 2005; [68]). Our dose is approximately 3.5 time the high end of the dose (80 mg/kg) for a 200 lb adult. Because zebra finches are smaller (~15 grams vs mouse ~20 g) and have a higher resting metabolic rate than mice, this is a conservative approximation of the human equivalence dose [69,70]. In other words, our dose in birds is a closer approximation to the FDA approved human dose for children than if we were using mice with a 50 mg/kg dosage.

### BrdU administration

To label mitotically active cells, birds received intramuscular injections of the thymidine analog BrdU three times a day for three consecutive days on days 30, 31, 32 prior to the day of perfusion, thereby marking neurons that were 30–32 days old at the time of perfusion. We used a dose of 0.074 mg BrdU (Sigma-Aldrich, B5002) per gram weight of bird, dissolved in tris buffered saline. Because neurogenesis is sensitive to social housing conditions [71] we allowed birds to acclimate to isolate housing in sound attenuated chambers for 30–60 days prior to the first BrdU injection. Birds were between 75–79 days old at the first BrdU injection and 77–81 days old at the last BrdU injection.

### Perfusion and histology

Birds were transcardially perfused at ages 107–111 days old (30 days after the last BrdU injection). The birds were deeply anesthetized with euthasol (Penn Vet Supply, PVS111) then transcardially perfused with 0.1 M phosphate buffered saline (PBS), followed by 4% paraformaldehyde (PFA). The brains were removed and fixed with 4% PFA for one hour then stored in PBS overnight at 4°C. Brains were divided into hemispheres, dehydrated in increasing concentrations of ethanol and embedded in polyethylene glycol (molecular weight 1500, Sigma-Aldrich, Polysciences, 25322-68-3). There is no evidence of a hemispheric difference in statins crossing the blood-brain barrier, therefore we arbitrarily selected the left hemisphere for processing. Left hemispheres were sagittally sectioned at 6 μm using a rotary microtome. Every eighth section was collected in three series, mounted onto Superfrost+ charged slides and stored at -20°C.

### Immunohistochemistry

One slide series was used to label BrdU and the neuron-specific protein Hu, which is expressed in the cytoplasm as a cell commits to a neuronal fate and is continually expressed throughout the neuron’s lifespan [72,73]. Slides were heated in citrate buffer for 10 min at 90–95°C then rinsed for 5 min in PBS. The slides were then immersed in a solution of 0.1 N HCl with 0.28% pepsin at 37°C for 3 min followed by three 5-min rinses in PBS. To block non-specific binding sites, slides were incubated for 1 hour in 0.3% Triton-X and 10% normal donkey serum in PBS (“blocking solution”). Tissue was incubated overnight at 4°C in anti-BrdU primary antibody made in sheep (1:194 in blocking solution, Capralogics, P00013, polyclonal). After three PBS washes of 10 min, 4 drops of avidin block (Invitrogen, R37627) were applied to each slide for 15 min. Slides underwent three 10-min PBS rinses followed by 4 drops of biotin block (Invitrogen, R37627) for 15 min. Tissue was then incubated for 1 hour at room temperature in streptavidin-conjugated Alexa 488 donkey anti-sheep IgG (1:800 in PBS, Thermofisher, S11223). The slides were rinsed three times for 10 min each in PBS then incubated for 1 hour in blocking solution. Primary antibody to Hu made in mouse (1:200 in PBS, Life Technologies, A-21271, clone 16A11) was then applied to slides and incubated overnight. Slides were rinsed in PBS three times for 10 min, then treated with donkey anti-mouse antibody conjugated to Cy3 for 1 hour (1:80 in blocking solution, Jacksonimmuno Research, catalog 715-165-150, polyclonal, RRID #AB_2340813). Slides were rinsed three times in PBS for 10 min, then submerged in deionized water for 30 sec. Finally, slides underwent dehydration in 50% ethyl alcohol (ETOH) for 30 sec, 70% ETOH for 30 sec, followed by 95% ETOH, 100% ETOH, and xylenes each for 1 min. After removal from xylenes, slides were cover slipped with Krystalon (Harleco, Sigma-Aldrich, 64969).

A second series of slides was used to label the early neuronal marker doublecortin (DCX) [74]. The slides were brought to room temperature and washed in tris buffered saline (TBS) for 10 min. Then they were incubated in a hydrogen peroxide solution (2% hydrogen peroxide, 97% TBS, 1% methanol) for 30 min to quench endogenous peroxidase. After three 5-min TBS rinses, slides were blocked with 3% normal horse serum and 2.5% Triton X-100 in TBS for 30 min at room temperature (“blocking solution”). The tissue was then incubated in primary anti-doublecortin made in goat (1:150, Santa Cruz Biotechnology, sc-8066, polyclonal) in blocking solution at 4 ºC for about 36 hours. On the third day, slides were rinsed with TBS three times for 5 min each, then 4 drops of avidin block (Invitrogen, R37627) were applied to each slide for 15 min. Slides underwent three 10-min TBS rinses followed by 4 drops of biotin block (Invitrogen, R37627) for 15 min, followed by three 10-min TBS washes. Tissue was then incubated with biotinylated horse anti-goat antibody in TBS (1:200, Vector Laboratories, BA-9500) for 3 hours. Slides were again rinsed in TBS three times for 5 min each, and then incubated in ABC solution prepared from the ABC Elite Kit (Vector Laboratories) for an hour. The slides were rinsed for 5 min three times with TBS, then incubated in diaminobenzidine (DAB) solution (Vector Laboratories). After three 5-min TBS rinses, sections were dehydrated as follows: 30 sec in deionized water, 1 min in 50% ETOH, 3 min in 70% ETOH, 5 min in 95% ETOH, two 10-min washes in 100% ETOH, and two 15-min washes in xylenes. The sections were cover slipped with Krystalon (Harleco, Sigma-Aldrich, 64969).

### Microscopy

#### Regions.

All microscopy was performed blind to bird identity and treatment. Brain tissue was observed using a computer-controlled fluorescence microscope (Olympus BX51). Mapping software (Lucivid microprojection and Neurolucida, Microbrightfield Bioscience Inc.) was used to trace the brain regions HVC and the caudomedial nidopallium (NCM), mark and quantify new neurons, trace neuronal somas, and produce soma feature measurements on closed contours.

**Fig 1:**
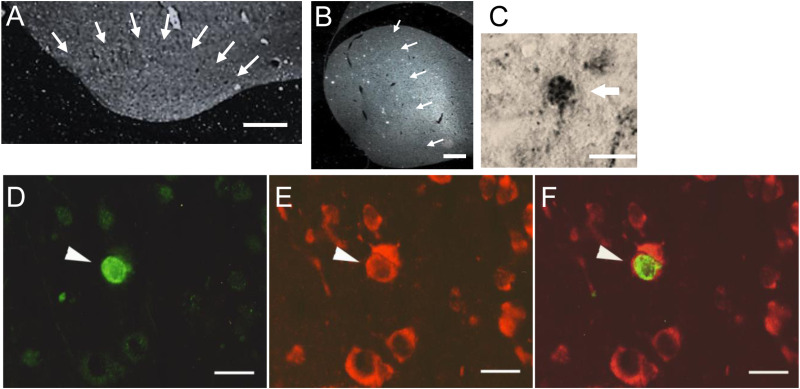
Region and cell microscopy. The internal boundaries of nucleus HVC (A) and region NCM (B) are approximated by arrows, shown in dark field. Field L region L2 can be seen as a bright band to the right (rostral) of the arrows in (B). Doublecortin+ neuron is shown labeled with DAB in HVC (C). BrdU-labeled cell shown visualized with a fluorescein isothiocyanate (FITC) filter (green, D) and Hu-labeled cells visualized with a rhodamine filter (red, E). Double-labeled cells were identified by alternating between these two filters and also using a dual FITC-rhodamine filter (F). Scale bars A = 300 µm, B = 1 mm; C = 20 µm, D-F = 10 µm.

HVC was identified by the location of the landmarks: hippocampus, cerebellum, and the HVC downstream target robust nucleus of the arcopallium (RA) under dark field optics (Fig 1A). The caudal and ventral edges of the telencephalon determine the caudoventral boundary of NCM [75]. The caudal medial mesopallium (CMM) is adjacent to the rostral edge of NCM in medial sections and demarcated by a visible lamina. In more lateral sections, the rostral border of NCM was defined as ~ 300 µm caudal to the Field L Complex subdivision L2, which is identified using dark field optics and appears as a diffuse bright band of densely packed cells rich in neuropil [76] as in [77] (Fig 1B).

HVC and NCM were selected for quantification of new neurons because there is extensive literature on neuronal recruitment and new neuron survival in these regions that underlie song learning and production. Therefore, the present work could lay the groundwork for studies of song learning behavior with known functional correlates of neurogenesis. Likewise, we focused on HVC for quantifying neuronal morphology because the neural populations in this nucleus are well described. We selected BrdU + /Hu + new neurons of known age in HVC to minimize morphological variability due to differences in neuron age. In particular, young neurons still expressing DCX+ have a wide range of variability in soma phenotype since some still exhibit a large variation in fusiform in shape while migrating yet others have begun to mature establish a roundish soma phenotype [74]. In addition, soma size of many neuronal cell types decreases with increasing cell age. Therefore, in order to limit neuron morphological variability due to cell age, we first measured morphological features of mature BrdU + /Hu+ cells, limiting our population to new neurons aged 30–32 days post mitosis.

In addition, we also sought to limit morphological variability due to differences in neuron types; therefore, we restricted our measurements to neurons in HVC only. Adult-born neurons in HVC are limited to two neuronal types: about half are RA-projecting neurons and half are DARPP32 + non-RA projecting neurons [61]. In contrast, new neuron cell types, and potential heterogeneity of the population of new neurons in NCM have not yet been studied. Therefore, to minimize morphological variance due to differences in cell ages and types, we first examined BrdU + /Hu+ neurons in HVC, born during statin administration. Although there is a mix of neuron types in HVC that would not be marked with BrdU, we continued our analysis to compare soma metrics of all nonBrdU-labeled neurons between statin-treated and control birds, to potentially identify any large scale or overarching changes in cell size or shape due to statins.

#### Quantification of new neurons.

DCX+ cells were identified using lightfield optics (Fig 1C). BrdU + /Hu+ cells (30–32 days old) were visualized by switching between the FITC and rhodamine filters and using a dual FITC/rhodamine filter (Figs 1D - 1F). We calculated the density of BrdU + /Hu + and DCX+ cells per mm^2^ in 10–12 sections of HVC evenly distributed throughout HVC (n = 6 statin-treated, n = 7 controls). DCX + and BrdU + /Hu+ cells were quantified in NCM in 10–12 sections from ~300–700 µm lateral to the midline (n = 5 statin-treated, n = 7 controls). Tissue from one statin-treated bird was lost during processing. Neuronal densities were calculated by dividing the total number of labeled cells summed across all sections by total area sampled summed across all sections. Cross-sectional areas of 20 HVC sections distributed evenly throughout HVC, including the most medial and lateral sections containing HVC were measured using Neurolucida and darkfield optics, multiplied by section thickness and the sampling interval to estimate total HVC volume. New neuron density was multiplied by total HVC volume per bird to estimate numbers of new neurons in the total HVC.

#### Soma feature measurements.

We were interested in whether neurons formed during statin exposure showed aberrant soma morphology. To do this, we traced soma contours of all BrdU + /Hu+ labeled cells in our samples of HVC (n = 6 statin-treated, 7 control birds; 13–100 cells per bird; mean = 40 cells per bird; SEM = 7.96) and compared contour morphometrics between statin-treated and control birds. Only two types of neurons are added to HVC in male zebra finches at the ages when BrdU was administered (75–81 days of age). The first project to RA and the second population do not project to RA and are characterized by their expression of the DARPP32; each contribute about 50% of the new added to HVC [61,78,79]. Therefore, our BrdU + /Hu+ cells consisted of these two neuron types and based on our 3 days of cell birthdating, were limited to neurons 30–32 days of age [61,80,81]. All neurons labeled with BrdU + /Hu+ were formed during exposure to statins, when birds were 75–81 days of age. Note, we are reporting BrdU+ labeled cell ages as 30–32 days, based on the 30 day survival time after the last BrdU injection and 3 days of BrdU injections. However stem cells in mammalian proliferative zone can incorporate BrdU and then delay mitosis, or undergo multiple symmetrical mitotic events, prior to producing migratory neuronal precursors [82]; therefore, it is possible our youngest BrdU+ neurons are less than 30 days old. Other work confirms that BrdU at the doses used here does not interfere with neurogenesis in the zebra finch [83].

Mature neurons continue to require local cholesterol for structural and functional maintenance [84]. Therefore, we were also interested in whether statin exposure in juveniles might alter soma morphology of mature neurons already established within HVC during statin treatment [80]. To test this, we traced the contours of a sample of HVC Hu+ neurons that were not dividing during BrdU injections and therefore did not contain BrdU (BrdU-/Hu+), and compared morphometrics of these cells between statin-treated and control birds.

To sample BrdU-/Hu+ cells, we placed a grid containing squares of 100 µm by 100 µm superimposed completely over each HVC tracing, and only BrdU-/Hu+ neurons from every fourth square within the grid were traced, sampling approximately a quarter of each HVC section. To avoid over sampling, cells touching the top and left sides of the grid were not traced [as in 85]. We traced 14–66 BrdU-/Hu+ cells per bird (mean = 38 cells/bird, SEM = 3.87, n = 6 statin-treated, n = 7 control birds). This heterogeneous population of BrdU-/Hu+ cells included Area X-projecting neurons, nucleus avalanche-projecting neurons, and multiple types of inhibitory interneurons [78,86], as well as RA-projecting and DARPP32 + neurons that were not labeled with BrdU [61]. Except for RA-projecting and DARPP32 + neurons that escaped BrdU labeling, BrdU-/Hu+ cells were formed *in ovo* and peri-hatching; therefore, the majority of cells in this population were older than the population of birthdated BrdU + /Hu+ cells [78,87–89].

Cell somas were traced using a 60x (1.4 NA, oil) objective and unitless feature measurements were automatically generated by Neurolucida, defined by the Neurolucida User’s Guide (http://www.mbfbioscience.com/) as follows.

Aspect ratio is a measure of flatness. Values close to 1.0 indicate contours resemble a circle, and values less than 1.0 indicate increasing ovality or flatness.


$$AspectRatio=\frac{MaximumDiameter}{MinimumDiameter}$$

Compactness is a measure of the relationship between a contour area and maximum diameter, with a value of 1.0 indicating a circle, which has a maximum area to perimeter relationship, whereas values 0.0–1.0 describe a non-circular smaller area compared with the perimeter.


Compactness=4πArea/FeretMax

The roundness parameter is a function of the contour compactness value, and similarly lies on a scale of 0.0 to 1.0, but differentiates contours with small compactness values.


$$Roundness=\frac{4\times Area}{\pi \times FeretMax^{2}}$$

Shape factor and form factor measure the complexity of a contour outline defined by the relationship between a contour perimeter and area. A large perimeter relative to area results in a large shape factor and indicates a convoluted outline. The minimal shape factor, indicating a smooth contour, is measured on a circle, with a value of ~ 3.54.


$$ShapeFactor=\frac{Perimeter}{\sqrt{Area}}$$

Form factor measures both the roundness and fine-scaled complexity of a perimeter. For instance, a circle with a smooth perimeter has a form factor of 1. A circle with a rough perimeter, increasing perimeter length relative to area, has a form factor less than 1. Form factor differs from shape factor by measuring roughness on a finer scale of jaggedness whereas shape factor measures larger convolutions.


$$FormFactor=\frac{4\pi Area}{Perimeter^{2}}$$

The maximum Feret diameter is the maximum distance between any two points on the contour, which necessarily encompass the contour. The minimum Feret diameter is the minimum distance between two parallel lines that encompass the contour.

### Statistics

To determine whether statin treatment affected densities of new neurons, we used two-tailed independent t-tests to determine whether there were differences between the statin-treated birds and the control birds in densities of new neurons (BrdU + /Hu + and DCX+) within HVC and within NCM. All statistics were conducted on measures of new neurons within the regions of HVC or NCM and not combined across regions, thus brain region was not included as a factor for any statistics. Likewise, we used two-tailed independent t-tests to compare each neuronal morphological measurement of 30–32 day old (BrdU + /Hu+) neurons, and all neurons (mostly pre-existing, putatively mature, BrdU-/Hu+) neurons in HVC between statin-treated and control birds. To determine whether statins differentially affected morphology of new neurons in HVC (BrdU + /Hu+) compared with all neurons in HVC (BrdU-/Hu+), we used a 2-factor (statin-treated versus control groups) repeated measures (measures of BrdU-/Hu+ cells and BrdU + /Hu+ cells within the same tissue sample) ANOVA to compare effects of statins on these 2 neuron populations followed by post-hoc paired 2-tailed t-tests. The comparisons of morphological features were corrected using the Benjamini and Hochberg false discovery rate.

We used linear regression analyses to determine whether the following variables predicted densities of new neurons labeled with BrdU or DCX in HVC or NCM: the bird’s age at the time of BrdU injection (for the BrdU+ cell values), the bird’s age at the start of treatment, duration of statin treatment, or the bird’s age at perfusion. We conducted these analyses within the statin-treated group, within the control group, and within both groups combined. We also used linear regression analyses to determine whether these same variables (bird age at BrdU injection, age at start of treatment, duration of statin treatment, and age at perfusion) predicted each of the morphological measures within the statin-treated birds. Values reported are means ± SEMs. The alpha level was set to 0.05 for all statistics.

## Results

### Bird ages and experimental parameters did not correspond to densities of new neurons or soma morphology

Numbers of new neurons recruited to HVC decrease with the age of the bird [90,91]. For this reason, we used a narrow (5-day) range of bird ages at time of BrdU injections, 75–79 days old at first injection, and all birds were perfused 30 days after the last injection to compare densities of same-aged (30–32 day old) BrdU-labeled neurons. As expected, there was no correlation between the bird’s age at time of BrdU injection over this narrow range and the densities of new neurons labeled with BrdU in HVC or NCM in controls, statin-treated, or all birds combined (p > 0.05 for all).

On the other hand, we used a wide (31-day) range for the age of the bird at the start of the statin/vehicle treatment (18–49 days) to test whether the age at treatment onset or duration of treatment impacts densities of new neurons. This range of treatment onset spans approximately half of the postnatal maturation period, with reproductive and singing maturity occurring at around 90 days of age [92]. We found no correlations between the age of the bird at start of treatment or duration of statin treatment and densities of new neurons in HVC or NCM (p > 0.05 for all). We also determined that bird age at perfusion was not correlated with densities of new neurons in HVC or NCM (p > 0.05 for each, S1 Table). Likewise, we also found that none of these experimental variables (age of the bird at start of treatment, duration of statin treatment, bird age at first BrdU injection, or bird age at perfusion) predicted any of the morphological metrics of new neurons or measures of all neurons sampled in HVC (p > 0.05 for each).

### Atorvastatin did not affect densities of new neurons or HVC volume

Treating cultured neurons with lovastatin has been shown to increase cell death after 2–12 days of exposure [50,52]. Therefore, we asked whether daily oral administration of atorvastatin prior to and during the time of neuronal mitosis and continuing for a month post-mitosis would decrease new neuron production or survival *in vivo.* Birds had been given daily doses of atorvastatin for an average of 42 days (SEM = 2.645, range 30–60) prior to cell birth-dating, and for 30 days after the last day of cell birth-dating until the day of perfusion.

**Fig 2:**
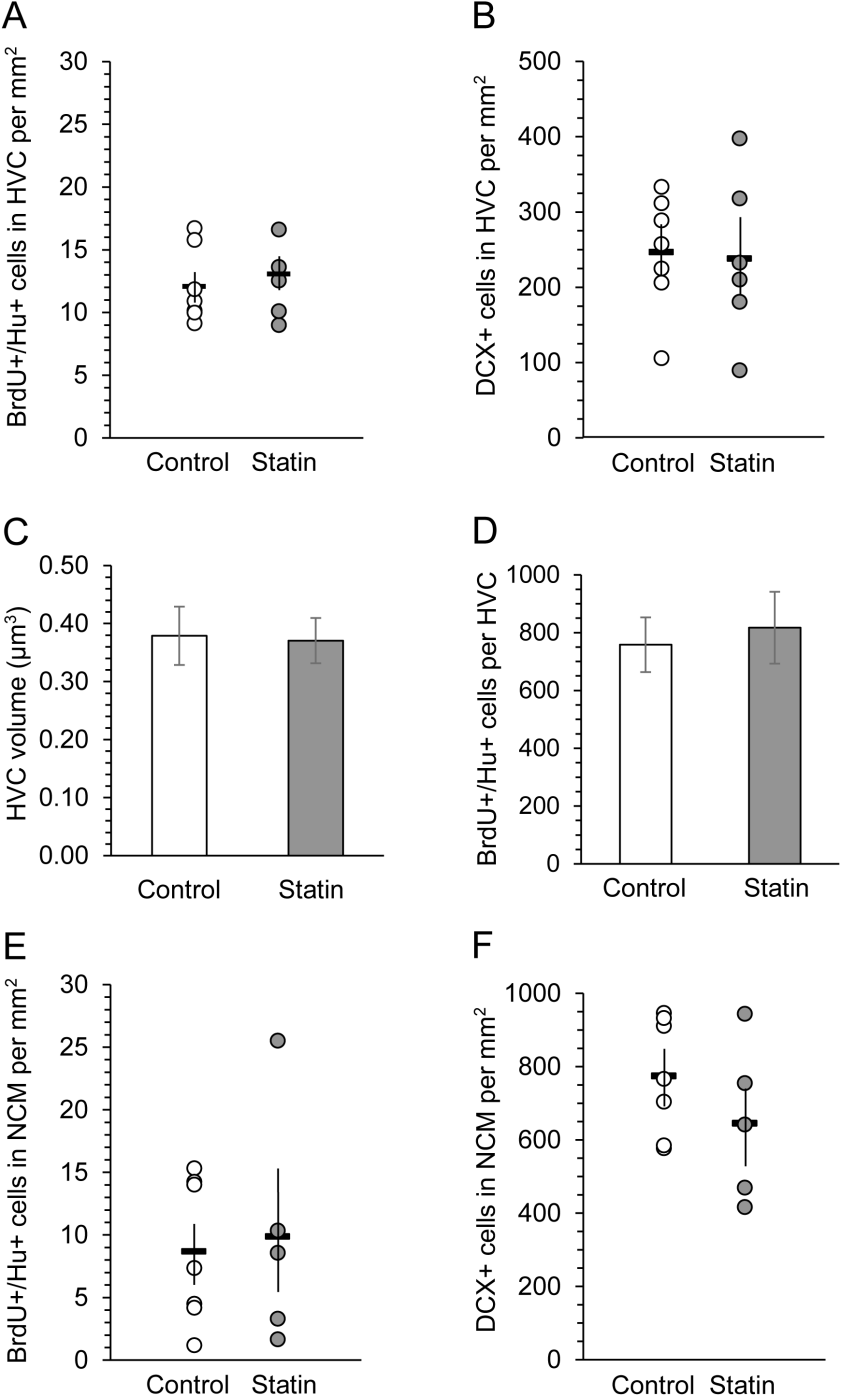
There was no effect of statins on numbers of new neurons. Densities of BrdU + /Hu+ cells (30 - 32 day old neurons) in HVC (A, control n = 7, statin-treated n = 6) did not differ between treatment groups. Densities of DCX+ cells (0 ~ 3 week old neurons) in HVC (B, control n = 7, statin-treated n = 6) also did not differ between treatment groups. There were no differences in estimated HVC volume (C) or estimated numbers of BrdU + /Hu+ cells per HVC between control birds and statin-treated birds (D). Densities of BrdU + /Hu+ cells (E) or DCX+ cells (F) in NCM also did not differ between the control and statin-treated groups (control n = 7, statin-treated n = 5). For A, B, E, F: Each marker is the mean number of cells per mm^2^ for an individual bird. Open dots = control birds, gray dots = atorvastatin-treated birds. Horizontal bars indicate the means of each group, vertical bars show SEM. C and D show means + /- SEMs.

We found no difference in the density of BrdU + /Hu+ neurons between atorvastatin-treated birds and controls in either HVC or NCM (HVC: t (11) = 0.588, p = 0.569; NCM: t (10) = -0.274, p = 0.789, Figs 2A and 1E). Estimated HVC volume did not differ between controls and statin-treated birds (t(11) = 0.141, p = 0.891, Fig 2C). Numbers of estimated BrdU + /Hu+ cells per HVC also did not differ between controls and statin-treated birds (t(11) = 0.382, p = 0.710, Fig 2D).

We also quantified DCX-immunoreactive cells to determine whether there were treatment differences in densities of new neurons in a younger neuronal cohort. Balthazart et al. [74] estimated that DCX is expressed in newborn neurons in the songbird for approximately 3 weeks, and if similar to mammals, expression begins during a proliferative progenitor cell stage [93]. The density of neurons that expressed doublecortin in HVC or NCM did not differ between the statin-treated and control birds (HVC: t (11) = 0.168, p = 0.869; NCM: t(10) = 1.200, p = 0.258; Figs 2B and F). Estimated numbers of DCX+ cells per total HVC likewise did not differ between controls (mean = 14,951, SEM = 1974) and experimental birds (mean = 14,389, SEM = 2810) (t(11) = 0.167, p = 0.870).

### Atorvastatin altered soma morphology

We hypothesized that statin treatment may disrupt the integrity of the neuronal membrane, perhaps affecting neuronal structure. Because we used thin sections, we focused on soma morphology rather than neurites. In order to minimize morphological variance due to multiple cell types, we limited our measurements to neurons in HVC which have been well characterized; less is known about the heterogeneity of neuron populations in NCM.

**Fig 3:**
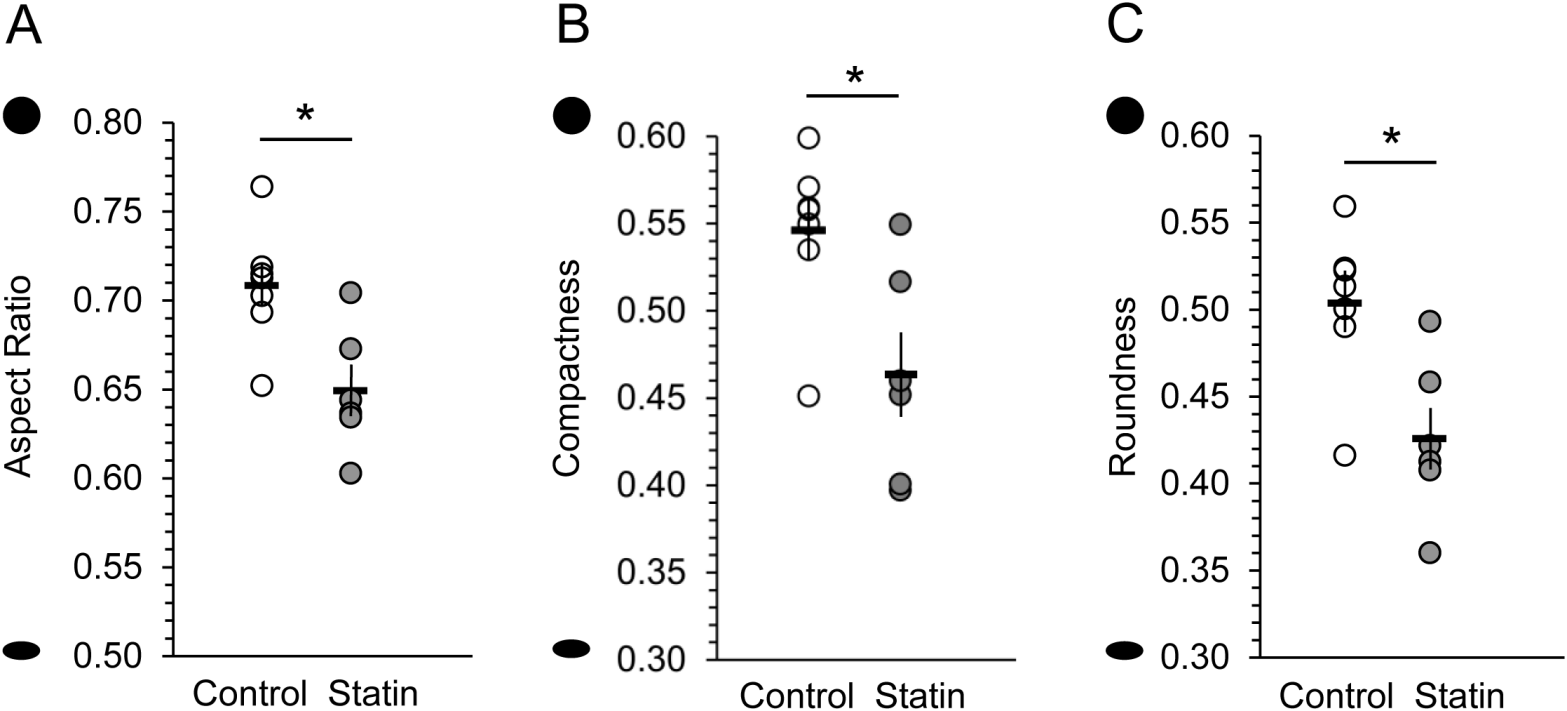
Statins induced flatter neuronal somas. In statin-treated birds, the somas of 30–32 day old (BrdU + /Hu+) neurons in HVC were flatter than those in controls as shown by significantly lower aspect ratio (A), compactness (B), and roundness (C). For all, a value of 1.0 describes a circle and lower numbers indicate increasing flatness. Each marker is the mean of cell measures for an individual bird. Horizontal bars indicate the means of each group, vertical bars show SEM. Y axis shapes indicate extreme contours for reference. Open dots = control birds, gray dots = statin-treated birds. * = p < 0.05.

We found that BrdU + /Hu+ neurons in statin-treated birds were flatter than those in the control birds as measured by: aspect ratio (t (11) = -3.11, p = 0.010, Fig 3A), compactness (t (11) = -2.800, p = 0.017, Fig 3B), and roundness (t (11) = -3.122, p = 0.010), Fig 3C). Roundness is derived from the compactness value and identifies differences in contours with small compactness values. With all 3 measures, a value closer to 1.0 describes a circle. Flatness measures are independent of perimeter complexity.

**Fig 4:**
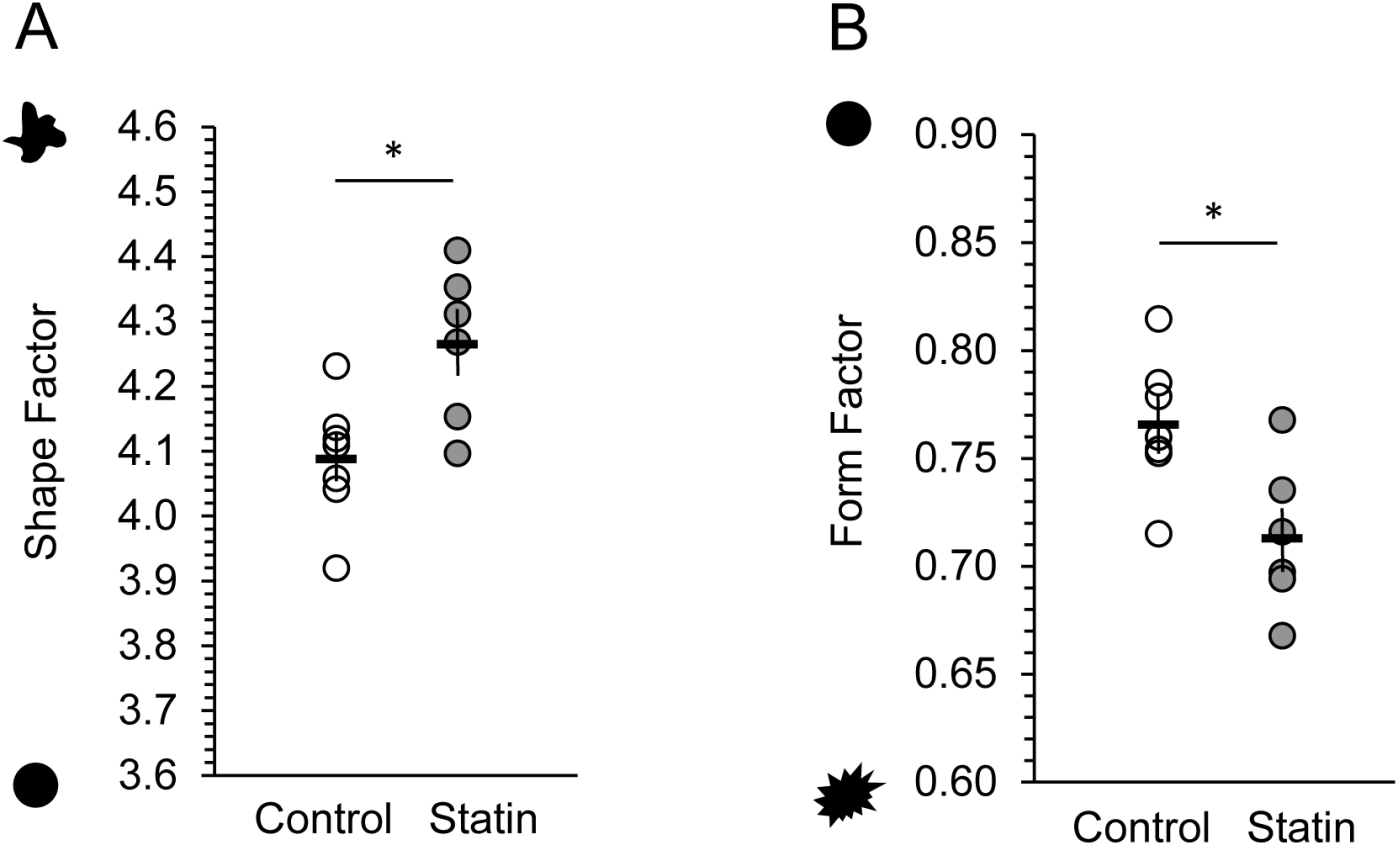
Statins increased neuronal soma membrane complexity. In statin-treated birds, the somas of 30 - 32 day old (BrdU + /Hu+) neurons in HVC had greater contour complexity than those in controls. Shape factor is a measure of the area to perimeter ratio of a contour (A), and form factor is a measure of compactness and contour complexity (B). Birds treated with statins had neuronal soma contours that were more convoluted as shown by higher shape factor values (A), and lower form factor values (B). Horizontal bars indicate the means of each group, vertical bars show SEM. Y axis shapes indicate extreme contour examples for reference. Open dots = control birds, gray dots = statin-treated birds. * = p < 0.05.

Perimeter complexity was measured by shape factor and form factor. These measures are complementary but differ in that shape factor considers a larger scale of surface jaggedness than does form factor–similar to the difference between surface foldings or convolutions versus surface pockmarks or roughness. Shape factor of new neurons was significantly higher in statin-treated than control birds, indicating a greater degree of membrane convolution (t (11) = -2.959, p = 0.013, Fig 4A). New neurons in the statin-treated birds also had a rougher membrane surface than in control birds, indicated by lower values of form factor (t (11) = 2.866, p = 0.015, Fig 4B).

**Fig 5:**
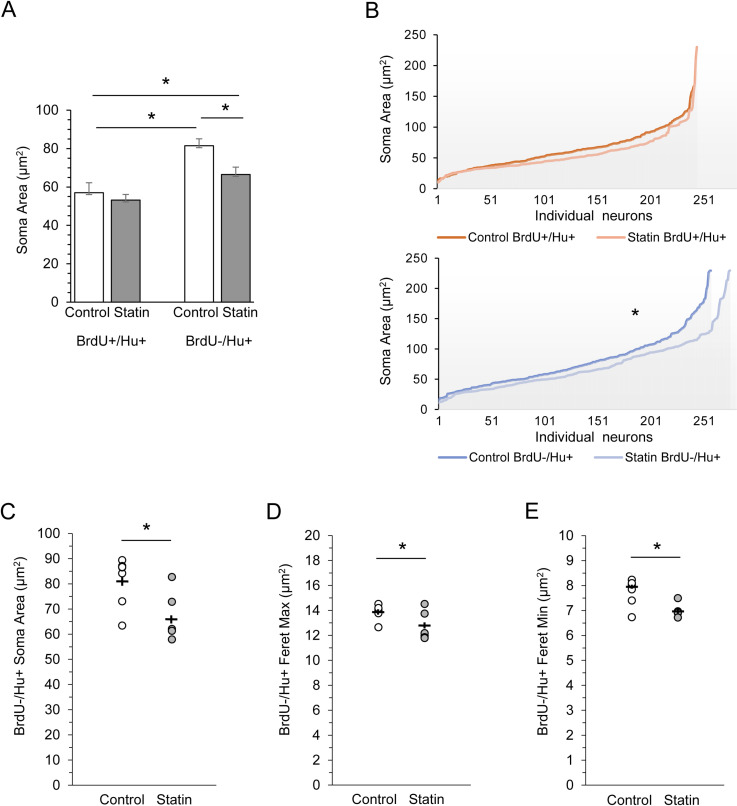
Statins resulted in smaller neuronal soma size. Soma areas of BrdU + /Hu+ (30 - 32 day old) neurons did not differ between control birds and statin-treated birds, whereas soma areas of the older heterogeneous population of BrdU-/Hu+ neurons were significantly smaller in statin-treated than in control birds (A). Within control birds, the BrdU + /Hu+ neuron population was significantly smaller in soma size than the BrdU-/Hu+ older, heterogeneous population (A). The same data as in (A) plotted by areas of individual neurons, across individuals, and ordered by ascending size along the X axis are shown in (B). Areas of all BrdU + /Hu+ neurons measured in control birds (dark orange line) and statin-treated birds (light orange line) shows qualitatively that statins did not decrease soma sizes of small cells (overlap of orange lines), and statins did not curtail the upper limit of soma size (light orange line reaches a soma size of > 200 µm, Y axis, top panel). Areas of cell somas in the heterogeneous BrdU-/Hu+ group are significantly smaller in the statin-treated (light blue line) group compared to soma areas in controls (dark blue line, bottom panel). Statin treatment decreased cell sizes in the larger cells more than in the smaller cells as seen in the greater divergence between lines plotted for larger cell somas (bottom panel). Soma areas of BrdU-/Hu+ neurons, (same data as BrdU-/Hu+ cells shown in A) plotted as means for individual birds, again show smaller sizes in statin-treated birds than in control birds (C). Consistent with this finding, the same neurons had a shorter Feret maximum (D) and Feret minimum diameter (E) in statin-treated birds than in controls. Horizontal bars indicate the means of each group, vertical bars show SEM. Open dots = control birds, gray dots = statin-treated birds. * = p < 0.05.

The overall size of BrdU + /Hu + new neuron somata in statin-treated birds did not differ from that of control birds in measures of area (t(11) = 0.584, p = 0.571; Figs 5A and 5B top panel), maximum Feret diameters (t(11) = 0.451, p = 0.661), or minimum Feret diameters (t(11) = 0.307, p = 0.764).

We next assessed the BrdU- heterogeneous population of Hu + HVC neurons, mostly older than the BrdU + /Hu+ cells and largely, but not exclusively, produced prior to statin treatment. HVC contains at least 5 neuronal types determined by electrophysiology, morphology, neurotransmitter, and projections [61,94–98], and 13 have been identified by gene expression profiles [99]. Using this large pool of neuronal types, we did not find a between-treatment difference in measures of flatness (aspect ratio, compactness, roundness) or shape complexity (shape factor and form factor) of BrdU-/Hu+ cells between statin-treated and control birds (p > 0.05 for all). Interestingly, we did find a treatment difference in soma size. Within the BrdU-/Hu+ neuron population, statin-treated birds had smaller soma sizes than control birds (t (11) = -2.836, p = 0.016, Figs 5A, 5B bottom panel, C). Consistent with this finding, the somas of BrdU-/Hu+ neurons in statin-treated birds had both shorter maximum Feret diameters and shorter minimum Feret diameters than BrdU-/Hu+ neurons in controls (t(11) = -2.476, p = 0.031, t(11) = -3.251, p = 0.008, respectively, Figs 5D and E5E).

It was intriguing that statin treatment did not affect soma size in 30–32 day old (BrdU + /Hu+) neurons, but resulted in decreased soma size in the heterogeneous non-birthdated population (Fig 5). This difference in outcome depending on our cell population could in principle be due to statins depressing the soma size of the BrdU- population, or increasing the soma size of the BrdU+ population. To identify the source of the effect, we directly compared soma areas between treatments (control and statin-treated) and neuron populations (BrdU+ versus BrdU- neurons). We found an overall effect of treatment such that statin-treated animals had a trend toward smaller neuron sizes (F(11) = 4.3, p = 0.062, with BrdU + and BrdU- neurons combined, Fig 5A). There was also a main effect of neuron population such that 30–32 day old neurons (BrdU + /Hu+) were significantly smaller than the heterogeneous, older population (BrdU-/Hu+) (F(11) = 25.15, p < 0.001, controls and statin-treated birds combined). There was no interaction between treatment and neuron population (F(1) = 2.09, p = 0.176).

Consistent with this, within control birds, soma sizes of the heterogeneous, older BrdU-/Hu+ neuron population was significantly larger than those of the 30–32 day old BrdU + /Hu+ neurons (post hoc paired 2-tailed t-test, t(6) = 4.951, p = 0.003). However, the statin-treated birds only showed a trend toward larger soma sizes of the heterogeneous, older neuron population compared to those of the 30–32 day old BrdU + /Hu+ neurons (post hoc paired t-test, t(5) = 2.203, p = 0.079). Thus, the effect of statin treatment was a significant decrease in size of neurons that were overall larger. Statins did not alter the overall range of cell sizes or limit the maximum soma size a neuron could attain (Fig 5B).

## Discussion

Products of the cholesterol biosynthesis HMG-CoA reductase pathway blocked by statins play essential roles in the proliferation and differentiation of neural progenitor cells *in vitro* [58,100]. Cell cultures have also demonstrated that decreasing cholesterol availability leads to membrane aberrations in newborn neurons [101]. Thus we hypothesized that newly formed neurons *in vivo* may similarly be sensitive to a decrease in cholesterol and other products of the HMG-CoA reductase pathway, reflected in new neuron numbers and morphology. Interestingly, we found no difference between statin-treated and control birds in densities of new neurons in two brain regions (HVC, NCM) sampled at neuronal ages 30–32 days or in a larger range of younger neurons approximately 0–3 weeks old. Together, this suggests that neuronal mitosis and early cell survival was not affected by 2–3 months of daily oral atorvastatin in juvenile zebra finches within our paradigm.

However, we found that statin treatment resulted in structural differences in HVC neuronal somata compared with neurons of control birds. In statin-treated birds, 30–32 day old (BrdU + /Hu+) neurons had flatter somata with more convolutions and a puckered surface compared to new neurons of the same age and cell types in control birds. Neurons that were not birthdated, putatively mostly older cells consisting of a heterogeneous population, did not show these effects of statins on neuronal soma shape or membrane surface, but were overall smaller in soma size in statin-treated birds than in controls. A potential explanation for the lack of effect on soma size in the BrdU + /Hu+ neuron population is that these neuron types (30–32 day old RA-projecting and DARPP32 + cells) are smaller than the mean soma size of the BrdU- neurons (Fig 5A) and therefore perhaps there is a floor effect beyond which statins cannot decrease soma size of viable neurons without affecting cell survival. In other words, perhaps the overall larger cell sizes of the heterogeneous population permitted a greater effect of statin-treatment on soma area.

The regulation and modulation of mevalonate pathway is well known to be sexually dimorphic [102] as are features of lipid metabolism and age-related changes in brain lipid composition [103]. Moreover, cholesterol is a precursor for the synthesis of various neurosteroids, which differ between males and females, as do their synthetic enzymes and transporters [104] and potential effects on adult neurogenesis [105]. Therefore, the relevance of our findings are restricted to males, a limitation of this study. It is critical to determine whether there are similar effects of statin exposure in females. It is also important to note that statins are generally prescribed to reduce pathological levels of cholesterol. In our experiments, we used statins without supplementing the animals with a high-cholesterol diet. Regardless, our manipulation is relevant to the long-running debate over administration of statins off-label and prophylactically under non-hypercholesterolemia conditions [18,106–110].

One model explaining our effects is that atorvastatin disrupted neural cholesterol production, decreasing cholesterol availability for appropriately structuring or distributing cholesterol throughout cell membranes, thereby affecting soma morphology. Atorvastatin has moderate lipophilicity and has been shown to cross the blood-brain barrier [111]. Numerous statins, including atorvastatin, reduce the cholesterol content of cultured neurons and glial cells [111]. Likewise *in vivo*, rats treated with either the lipophilic lovastatin or hydrophilic pravastatin had decreased total cholesterol in brain tissue [112], as did rodent models treated with lipophilic simvastatin or atorvastatin (review, [13]). In other work, atorvastatin, lovastatin, and simvastatin administered orally to mice resulted in CNS cholesterol redistribution in the inner and outer layers (leaflets) that make up the synaptic plasma membrane bilayer [8]. The synaptic plasma membrane cytofacial leaflet (cytosol-side of the bilayer) contains over 85% of the total synaptic plasma membrane cholesterol compared with the exofacial leaflet that is the extracellular membrane surface [113]. All three statins caused CNS cholesterol to translocate from the cytofacial leaflet to the exofacial leaflet [8]. At the doses used (50 mg/kg daily for 21 days), lovastatin significantly reduced total cholesterol *in vivo* in isolated plasma membranes [8].

Altered membrane cholesterol (in amount and placement) has been shown to result in detectable changes in cell structure [114–118]. For instance, in cultured hippocampal neurons, reduction of membrane cholesterol by incubation with methyl-β-cyclodextrin (MβCD), a cholesterol-binding agent, caused cell membranes to become less rigid [119]. A similar effect in our study may provide an explanation for the increase in perimeter complexity (convolutions and dimpling) as well as flattening of somas found in statin-treated birds. The amount of constituent membrane cholesterol has also been shown to affect the cell membrane and cytoskeleton interaction [120], which can alter cellular morphology as well. Cultured bovine aortic endothelial cells that were incubated in MβCD were smaller and thicker than control cells [120]. This is consistent with our finding of smaller, older neurons in our statin-treated birds, and suggests the idea that the shrinkage of older neurons in statin-treated birds may be caused by changes in the cell membrane and cytoskeleton interaction.

Independent of the plasma membrane, both microtubules and actin components of the cytoskeleton in neurons can be affected by statin exposure. Therefore, it is possible that the morphological effects seen in our study may be due to the breakdown of cytoskeleton, either solely or in addition to membrane dysfunction. In statin-treated cultured cells, microtubules found in the soma and cytoskeletal actin were fragmented and depolymerized [121]. These effects on the cytoskeleton involved the loss of function of microtubule-associated protein 2 (MAP2) and a neuro-cytoskeletal protein (NF200) [122]. Moreover, subsequent work found that it was the application of geranylgeranyl diphosphate (GGPP), an intermediate in the HMG-CoA reductase pathway that is blocked by statins, and not cholesterol, that reversed changes in microtubules and microfilaments of statin-treated neurons [123]. It is important to determine whether *in vivo* exposure to statins breakdown these cytoskeletal components, in turn destabilizing soma morphology. Actin assembly is also critical for neuronal maturation, which is marked by the extension of axons and dendrites [124]. Although we did not process the tissue in thick enough sections to determine whether statin treatment affected axons and dendrites, perhaps an interplay between stunted neurite growth and soma size contributed to our finding of smaller somas in the BrdU-/Hu+ neuron population.

An important caveat of our findings is that the observed effects of statins on soma size, shape, and membrane surface may be due to our histological processing essentially revealing differences in membrane composition. Our tissue was dehydrated with an ascending concentration series of ethanol and immersed in xylenes prior to coverslipping. Although these incubations were short (1 minute in 100% ethanol and 1 minute in xylenes) dehydrating and defatting tissue dissolves membrane cholesterol and displaces lipid rafts [125]. Therefore, differences in plasma membrane cholesterol composition between the control and statin-treated groups may have resulted in differential effects of ethanol and xylene, resulting in treatment differences in cell morphology that were unmasked or enhanced by these processing steps, and may not be apparent in tissue in natural extracellular fluid.

Based on findings of statin-induced decreased neuronal [58,100,101] proliferation and increased cell death in culture, we also hypothesized statin treatment may decrease neurogenesis *in vivo*. Contrary to this expectation, however, we found that daily doses of atorvastatin did not alter the density of new neurons in the zebra finch—a prolific model system sensitive to environmental effects on new neuron production and survival [126,127].

An interesting paradox of statins is that they are well known to be neuroprotective in models of disease, stroke, trauma, and high fat diet [128,129] and within these environments have been shown to suppress apoptosis [130–134], inflammation [130,134], and oxidative stress [135], and to increase neurogenesis and neuron survival in models of injury and impaired brain function [14,136,137]. Until recently, it had been suggested that neuroprotective effects of statins might require an impaired brain. However, Robin et al. [138] report increased neuronal proliferation *in vivo* in the healthy mouse brain following simvastatin treatment (10 mg/kg simvastatin by oral gavage daily for 7 days). This dose corresponds to an equivalent 5 mg/kg of atorvastatin and is comparable to 8.1 mg/kg simvastatin in humans, whereas the usual daily dose of simvastatin for adults is between 10 mg - 40 mg (per person regardless of weight) (https://www.fda.gov/media/72309).

Robin et al. [138] showed no effect of simvastatin on immature neuron numbers identified by doublecortin (DCX+), consistent with our findings using atorvastatin. However, after pulse labeling mitotically active cells 24 hours prior to perfusion (using 5-ethynyl-2′-deoxyuridine, EdU), they found significantly more DCX + /EdU+ cells in the dentate gyrus of simvastatin-treated mice indicating at least a transitory effect of simvastatin in increasing neuron proliferation [138]. In contrast, statins impaired neuronal survival in healthy neuron and glia cells in culture [56,139]. Thus it is possible that statins increase proliferation followed by increased new neuron death. Our results do not exclude the possibility of the same paired effect in our study as we found that atorvastatin did not change the net sum of neuron proliferation and new neuron survival within the first month post mitosis; however, we do not have a breakdown of proliferation and survival. It is possible that older neurons (BrdU-/Hu+) we measured in the statin-treated birds were in the process of dying as indicated by the size reduction of the soma, and that our size data captured this effect. Moreover, we did not quantify numbers of older neurons or the total neuronal density. Robin et al. [138] found no difference in apoptosis between statin-treated and control animals, assessed by caspase 3 activation. If there was similarly no difference in apoptosis in our study, this would suggest the size reduction of the soma following statin treatment need not indicate neuronal loss although we cannot rule it out.

While it is tempting to ascribe our findings of altered neuronal soma morphology to decreased cholesterol availability, it is important to keep in mind that statins are competitive inhibitors of HMG CoA reductase, an early enzyme of the cholesterol biosynthesis pathway that catalyzes mevalonate. As a result, the production of all intermediate and end products of the mevalonate pathway is diminished by statin exposure. Particularly relevant to our findings, statins also block the production of isoprenylated proteins, many of which are GTPases that bind and hydrolyze GTP. In particular, the Rho family of GTPases regulates numerous aspects of intracellular actin dynamics and cell structural morphology [140,141]. In addition, the Ras family of GTPases is critical for cell growth. In general, isoprenoids are compounded into numerous products that play a role in cell growth, differentiation, and cytoskeletal function [140]. Thus a decrease in any of these products may have contributed to our findings.

It must also be considered that statins may be indirectly affecting neuronal structure. Neuronal morphology may be influenced by the composition of the neuronal environment, as evidenced by effects of matrices, perineuronal nets, neuropil, and the chemical environment on neuronal shape during migration, maturation, and the establishment of processes [142]. In particular, the effects of atorvastatin on neuronal soma morphology in our study may also be an indirect consequence of statins interfering with any of these substrates and or an effect of statins on glia. In particular, microglia, astrocytes and oligodendrocytes are well known to impact neuronal function, including influencing neuronal metabolic processes, neurotransmitter cycling, signal transmission; providing trophic factors, regulating environmental homeostasis, and modulating inflammatory responses [143–145], which may in turn may potentially affect neuronal soma size and structure. Microglia and astrocytes also play a direct role in synapse formation and synaptic pruning [144,146] with the potential to impact neuronal soma morphology.

In sum, our results on altered cell structure provide a cautionary note about statin effects on cell structure in the brain *in vivo*, and should stimulate work that examines the microstructure of neural and other mitotically active cells (e.g., astrocytes and oligodendrocytes) in models of statin use. Additional next steps include determining whether changes in membrane morphology and overall soma size have physiological implications for neuronal and circuit behavior, whether statins impact channel density, distribution, or properties [147], as well as dendritic and axonal structure and function [148], and ultimately whether morphological effects on neural substrates can be linked to cognitive and/or behavioral effects potentially explaining complaints of memory impairment and brain fog by some statin users [31].

## Supporting information

S1 Table Linear regressions between experimental variables and new neurons in HVC and NCM. (PDF)
