# Supplementary material for: Daily high doses of atorvastatin alter neuronal morphology in a juvenile songbird model
Source: PLoS One. 2025 Apr 28;20(4):e0314690. doi: 10.1371/journal.pone.0314690 (PMC12036933; doi:10.1371/journal.pone.0314690)
Supplement: S1 Table — (PDF) [file pone.0314690.s001.pdf]

Supplemental Data Table 1. Values for linear regressions between experimental variables and new neurons in HVC and NCM. None are significant.

**Statin-treated Birds Experimental Variables**	**Cell Type (density per mm^2)**	R^2	**F**	**P**
Age of bird at start of treatment	BrdU+/Hu+ cells in HVC	0.152	0.716	0.445
	BrdU+/Hu+ cells in NCM	0.169	0.612	0.491
	DCX+ cells in HVC	0.151	0.712	0.446
	DCX+ cells in NCM	0.504	3.045	0.179
Duration of statin treatment	BrdU+/Hu+ cells in HVC	0.121	0.553	0.498
	BrdU+/Hu+ cells in NCM	0.131	0.451	0.550
	DCX+ cells in HVC	0.220	1.128	0.348
	DCX+ cells in NCM	0.457	2.524	0.210
Age of bird at first BrdU injection	BrdU+/Hu+ cells in HVC	0.146	0.683	0.455
	BrdU+/Hu+ cells in NCM	0.104	0.349	0.596
	DCX+ cells in HVC	0.157	0.743	0.437
	DCX+ cells in NCM	0.078	0.253	0.650
Age of bird at perfusion	BrdU+/Hu+ cells in HVC	0.146	0.683	0.455
	BrdU+/Hu+ cells in NCM	0.104	0.349	0.596
	DCX+ cells in HVC	0.157	0.743	0.437
	DCX+ cells in NCM	0.078	0.253	0.650
